# The dove of peace is not peaceful : the ghost of aortic arch pseudoaneurysm

**DOI:** 10.1186/s13019-026-04078-w

**Published:** 2026-04-14

**Authors:** Zixiong Nie, Longrong Bian, Honghua Yue, Weitao Liang, Zhong Wu

**Affiliations:** Department of Cardiovascular Surgery, West China Hospital, Sichuan University, Chengdu, China

**Keywords:** Cryptococcus, Infectious pseudoaneurysm, Aortic arch, Pigeon exposure, Endovascular stent graft

## Abstract

**Background:**

Infectious pseudoaneurysms are rare, and those caused by Cryptococcus, an organism commonly found in pigeon feces, are exceptionally uncommon. This case highlights this severe vascular complication as a sequel of cryptococcal infection.

**Case presentation:**

A 71-year-old male with a history of pigeon exposure presented with a persistent fever and was diagnosed with cryptococcal pneumonia and bacteremia. After successful initial antifungal treatment, a follow-up chest CT scan three months later revealed a pseudoaneurysm at the aortic arch. The patient underwent successful endovascular repair with a covered stent, achieving a satisfactory outcome.

**Conclusions:**

This case demonstrates that cryptococcal infection can lead to the serious complication of an aortic pseudoaneurysm, which may develop or be detected after the initial infection. It underscores the importance of long-term monitoring in patients with cryptococcemia and highlights the need for awareness of this risk in individuals exposed to pigeons.

**Supplementary Information:**

The online version contains supplementary material available at 10.1186/s13019-026-04078-w.

## Background

Infectious pseudoaneurysms represent an uncommon yet clinically significant vascular complication. While multiple pathogens have been associated with this condition, Cryptococcus stands out as an exceptionally rare etiology [1, 2]. Of particular epidemiological relevance is the well-documented presence of Cryptococcus in pigeon excreta, establishing avian exposure as a recognized risk factor for human infection.

## Case presentation

A 71-year-old male with no significant comorbidities presented with a persistent fever lasting two weeks. A chest computed tomography (CT) scan revealed extensive pulmonary infiltrates consistent with severe pneumonia, along with evidence of disseminated cryptococcemia. Subsequent blood analysis confirmed Cryptococcus infection.

Notably, the patient reported close contact with pigeons—specifically feeding them—during the two weeks preceding symptom onset. Following diagnosis of cryptococcal infection, he was started on a 2-week induction course of amphotericin B plus flucytosine. His clinical symptoms improved, blood cultures became negative, and he was subsequently discharged.

The patient was then transitioned to oral fluconazole for an additional 8 weeks as consolidation therapy before discontinuation. During a routine three-month follow-up, imaging unexpectedly identified a 2.7 × 3.9 cm pseudoaneurysm originating from the aortic arch. Given his well-controlled infection and advanced age, a multidisciplinary team (MDT) recommended thoracic endovascular aortic repair (TEVAR).

**Fig. 1:**
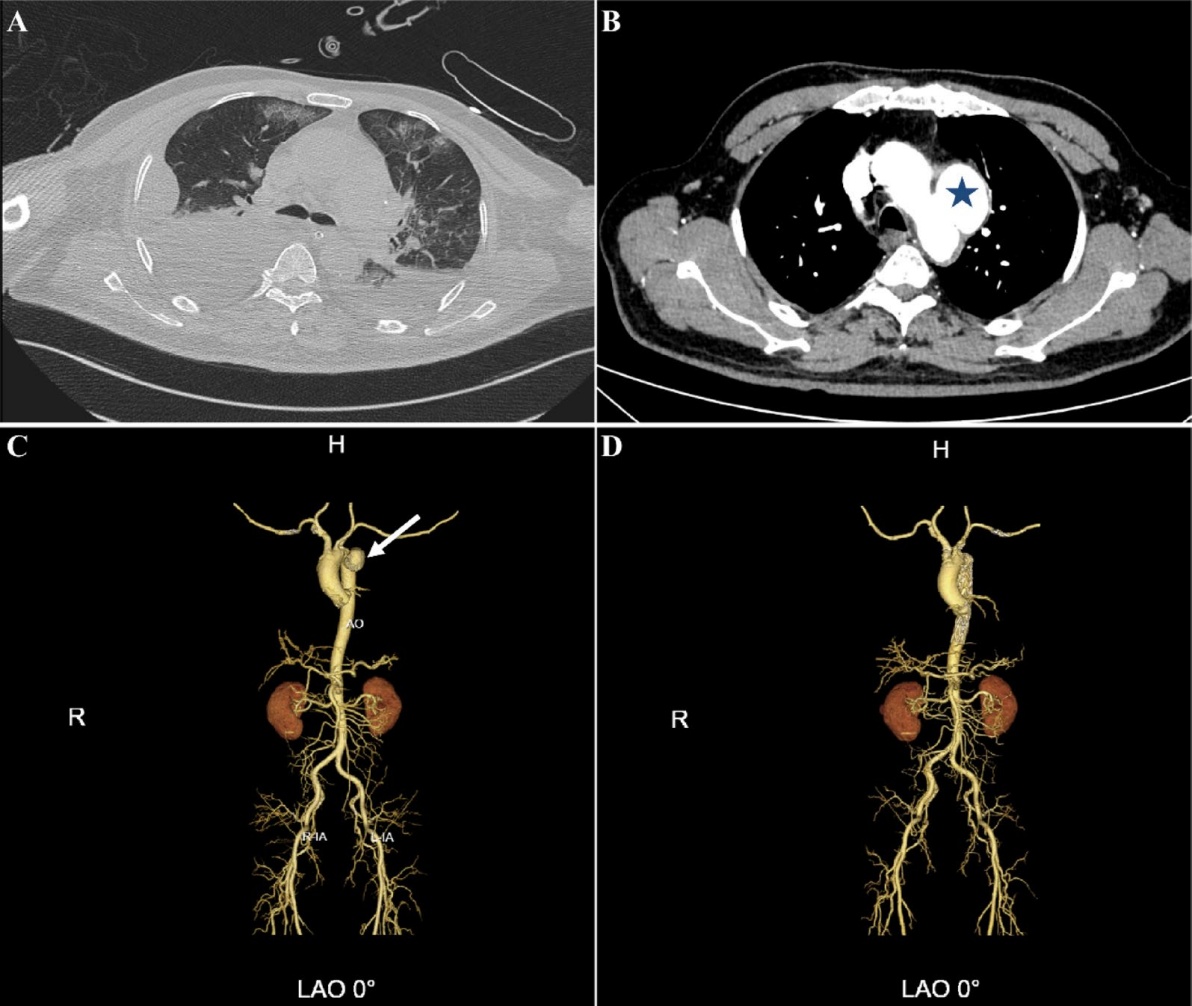
**A**, Chest CT shows severe lung infection and pleural effusion; **B**, The blue asterisk marks the location of the aortic arch pseudoaneurysm; **C**, Three dimensional reconstruction CT showed that the pseudoaneurysm was located in the aortic arch; **D**, Endovascular aortic intervention isolated the pseudoaneurysm.

The patient subsequently underwent successful endovascular exclusion of the pseudoaneurysm using a covered stent graft. Postoperative imaging confirmed technical success of the procedure (Fig. 1).

Video 1: Preoperative aortography showed that the pseudoaneurysm was located in the aortic arch.

Video 2: After stenting of the descending aorta, the aortic arch pseudoaneurysm was completely isolated, and no internal leakage was found.

## Discussion and conclusions

Pseudoaneurysm is a vascular disorder characterized by an incomplete arterial wall, which can lead to catastrophic consequences if not promptly and accurately managed. Pseudoaneurysms form as a result of disrupted arterial wall continuity, with blood extravasating into the surrounding tissues to form a blood-filled saccular structure adjacent to the damaged artery. This structure is typically eccentric and maintains continuous communication with the arterial lumen. The cystic lesion may be encapsulated by the tunica media or adventitia of the artery, or solely by the soft tissue structures surrounding the injured vessel [3].

This clinical report describes an unusual case of Cryptococcus-associated fungal pseudoaneurysm affecting the aortic arch, emerging as a delayed complication despite initial treatment response. A comprehensive discussion of this case involves the environmental factors of cryptococcal infection, the pathogenic mechanism of fungal infection-induced aortic pseudoaneurysm, surgical decision-making, and the clinical implications derived therefrom.

The main environmental exposure for cryptococcal infection is inhalation of contaminated aerosols, with common sources including pigeon droppings, decaying wood, and soil rich in organic matter [4]. High-risk activities associated with cryptococcal infection include contact with poultry, gardening, and forestry work—consistent with the present case, where the patient had close contact with pigeons (specifically feeding them) for two weeks prior to the onset of initial infectious symptoms. Notably, cryptococcal infection is more common in immunocompromised hosts; however, the present patient had no significant comorbidities, which makes this case even more distinctive. Therefore, when collecting clinical history from patients with suspected or confirmed systemic fungal disease, it is crucial to specifically inquire about the above-mentioned environmental exposure history, as a thorough environmental exposure history can provide valuable clues for etiological diagnosis and subsequent management. From a public health perspective, these findings further underscore the importance of risk awareness among individuals with significant avian exposure (Fig. 1).

Fungal pseudoaneurysms typically originate from infective arteritis or result from arterial wall thinning and destruction caused by inflammation induced by infectious agents. The underlying pathogenic mechanism involves microbial hematogenous dissemination to the vasa vasorum of the arterial wall, which elicits inflammation. This inflammatory response in turn destroys the adventitia and muscular layer of the arterial wall, further weakens its structural integrity, and ultimately leads to arterial wall rupture with invasion of surrounding tissues, resulting in a localized rupture and subsequent formation of a fungal pseudoaneurysm. In the present case, the patient had a documented history of cryptococcal infection, which is most likely the microbial etiology of the patient’s aortic arch fungal pseudoaneurysm. It is worth noting that among the pathogens causing fungal pseudoaneurysms, approximately 55% are Gram-positive cocci, of which Staphylococcus aureus accounts for roughly 45% and Streptococcus accounts for about 10%. Although abdominal aortic aneurysms secondary to cryptococcal infection have been reported in the literature, aortic arch fungal pseudoaneurysms associated with cryptococcal infection are relatively rare, which adds to the clinical significance of this case [5–7].

The management of fungal aortic pseudoaneurysms requires careful consideration of multiple factors, including infection control, patient age, comorbidities, and the location and size of the pseudoaneurysm. Traditionally, the standard therapeutic regimen involves open surgical repair, which consists of extensive debridement and resection of the infected aorta along with the surrounding contaminated tissues. This is followed by vascular reconstruction using either in-situ or extra-anatomic grafts, combined with long-term antibiotic therapy. However, previous reports indicate that this open surgical repair approach is associated with high mortality rates, likely due to multiple factors including the complexity of the procedure, severe comorbidities, and unstable patient conditions resulting from sepsis or arterial rupture [8].

In recent years, endovascular stent-graft repair has emerged as a feasible and less invasive alternative for the management of various aortic pathologies. Endovascular aortic repair (EVAR) has demonstrated favorable outcomes in the treatment of abdominal aortic aneurysms, with reduced perioperative mortality and lower complication rates. Furthermore, endovascular repair has been successfully applied in the treatment of fungal aortic pseudoaneurysms. In the present case, considering that the patient’s infection was well-controlled and he was of advanced age (71 years old) without significant comorbidities, a multidisciplinary team (MDT) discussion was conducted to weigh the risks and benefits of open surgical repair versus endovascular repair. Ultimately, the patient successfully underwent thoracic endovascular aortic repair (TEVAR) with a covered stent graft, and postoperative imaging examinations confirmed the technical success of the procedure. This choice of endovascular repair aligns with the trend of minimally invasive treatment for aortic pathologies in elderly patients, effectively reducing perioperative trauma and improving prognosis.

Several important clinical observations and implications emerge from this case. First, Cryptococcus demonstrates the potential to provoke vascular pathology (i.e., aortic fungal pseudoaneurysm) even after apparent control of the primary cryptococcal infection, highlighting the need for long-term follow-up of patients with documented cryptococcal infection. Second, the case reinforces the value of obtaining a thorough environmental exposure history in patients with systemic fungal disease, as environmental exposure (such as pigeon contact in this case) is often a key clue for etiological diagnosis. Finally, it raises the question of whether patients with documented cryptococcemia might warrant periodic vascular surveillance to detect potential delayed vascular complications at an early stage, thereby improving clinical outcomes.

## Supplementary Information

Below is the link to the electronic supplementary material.

Supplementary Material 1.

Supplementary Material 2.

Supplementary Material 3.

## Data Availability

Data sharing is not applicable to this article as no datasets were generated or analysed during the current study.

## References

[1] Deitch JS, Plonk GW, Hagenstad C, Hansen KJ, Peacock JE, Ligush J. Cryptococcal aortitis presenting as a ruptured mycotic abdominal aortic aneurysm. J Vasc Surg. 1999;30(1):189–92. 10.1016/s0741-5214(99)70191-6. 10394169 10.1016/s0741-5214(99)70191-6

[2] Karam J, Tsiouris A, Vazquez J, Shepard A. Cryptococcal aortitis presenting as a symptomatic abdominal aortic aneurysm. Ann Vasc Surg. 2015;29(2):363e. 9-363.e11. 10.1016/j.avsg.2014.08.025 25452084

[3] Jesinger RA, Thoreson AA, Lamba R. Abdominal and pelvic aneurysms and pseudoaneurysms: imaging review with clinical, radiologic, and treatment correlation. Radiogr: Rev Publ Radiol Soc N Am Inc. 2013;33(3):E71–96. 10.1148/rg.333115036. 10.1148/rg.333115036 23674782

[4] Iyer KR, Revie NM, Fu C, Robbins N, Cowen LE. Treatment strategies for cryptococcal infection: challenges, advances and future outlook. Nat Rev Microbiol. 2021;19(7):454–66. 10.1038/s41579-021-00511-0. 33558691 10.1038/s41579-021-00511-0 PMC7868659

[5] Berchtold C, Eibl C, Seelig MH, Jakob P, Schönleben K. Endovascular treatment and complete regression of an infected abdominal aortic aneurysm. J Endovasc Ther: Off J Int Soc Endovasc Spec. 2002;9(4):543–8. 10.1177/152660280200900426. 10.1177/152660280200900426 12223018

[6] Stanley BM, Semmens JB, Lawrence-Brown MMD, Denton M, Grosser D. Endoluminal repair of mycotic thoracic aneurysms. J Endovasc Ther: Off J Int Soc Endovasc Spec. 2003;10(3):511–5. 10.1177/152660280301000316. 10.1177/152660280301000316 12932162

[7] Aftab S, Uppaluri SAS. Mycotic pseudoaneurysm of the aortic isthmus secondary to salmonella infection causing a diagnostic dilemma. J Radiol Case Rep. 2019;13(4):17–27. 10.3941/jrcr.v13i4.3571. 31565178 10.3941/jrcr.v13i4.3571 PMC6743641

[8] Ting ACW, Cheng SWK, Ho P, Poon JTC. Endovascular stent graft repair for infected thoracic aortic pseudoaneurysms–a durable option? J Vasc Surg. 2006;44(4):701–5. 10.1016/j.jvs.2006.05.055. 16930927 10.1016/j.jvs.2006.05.055

